# ESCRT‐II functions by linking to ESCRT‐I in human immunodeficiency virus‐1 budding

**DOI:** 10.1111/cmi.13161

**Published:** 2020-02-14

**Authors:** Bo Meng, Natasha C Y Ip, Truus E M Abbink, Julia C Kenyon, Andrew M L Lever

**Affiliations:** ^1^ Department of Medicine University of Cambridge, Addenbrooke's Hospital Cambridge UK; ^2^ Department of Microbiology and Immunology National University of Singapore Singapore; ^3^ Homerton College Cambridge UK; ^4^ Department of Medicine National University of Singapore Singapore

**Keywords:** budding, ESCRT, HIV, late domains, maturation

## Abstract

Human immunodeficiency virus (HIV) uses the ESCRT (endosomal sorting complexes required for transport) protein pathway to bud from infected cells. Despite the roles of ESCRT‐I and ‐III in HIV budding being firmly established, participation of ESCRT‐II in this process has been controversial. EAP45 is a critical component of ESCRT‐II. Previously, we utilised a CRISPR‐Cas9 EAP45 knockout cell line to assess the involvement of ESCRT‐II in HIV replication. We demonstrated that the absence of ESCRT‐II impairs HIV budding. Here, we show that virus spread is also defective in physiologically relevant CRISPR/Cas9 EAP45 knockout T cells. We further show reappearance of efficient budding by re‐introduction of EAP45 expression into EAP45 knockout cells. Using expression of selected mutants of EAP45, we dissect the domain requirement responsible for this function. Our data show at the steady state that rescue of budding is only observed in the context of a Gag/Pol, but not a Gag expressor, indicating that the size of cargo determines the usage of ESCRT‐II. EAP45 acts through the YPXL‐ALIX pathway as partial rescue is achieved in a PTAP but not a YPXL mutant virus. Our study clarifies the role of ESCRT‐II in the late stages of HIV replication and reinforces the notion that ESCRT‐II plays an integral part during this process as it does in sorting ubiquitinated cargos and in cytokinesis.

## 1 INTRODUCTION

Endosomal sorting complexes required for transport (ESCRTs) are a family of cellular proteins comprising ESCRT‐0, ‐I, ‐II, and ‐III complexes and have been reviewed extensively (Olmos & Carlton, 2016; Schöneberg, Lee, Iwasa, & Hurley, 2016; Scourfield & Martin‐Serrano, 2017). Each is composed of two or more proteins: ESCRT‐0 (HRS and STAM complexes), ESCRT‐I (TSG101, VPS28, VPS37, and MVB12/UBAP1), ESCRT‐II (EAP20, EAP30, and EAP45), and ESCRT‐III (CHMP1–7) being the major recognised components. ESCRT proteins are classically involved in MVB formation (Katzmann, Babst, & Emr, 2001) and sorting of ubiquitinated cargos into vesicles involving sequential recruitment of ESCRT‐0, ‐I, ‐II, and ‐III. In addition to multivesicular body (MVB) formation, ESCRT complexes are involved in a variety of cellular functions including cytokinesis (Carlton & Martin‐Serrano, 2007), the biogenesis of microvesicles and endosomes (Nabhan, Hu, Oh, Cohen, & Lu, 2012), nuclear envelope resealing (Olmos, Hodgson, Mantell, Verkade, & Carlton, 2015; Vietri *et al*., 2015), membrane wound repair (Jimenez et al., 2014), neuron pruning (Zhang *et al*., 2014), and endolysosomal repair (Skowyra, Schlesinger, Naismith, & Hanson, 2018). The AAA ATPase VPS4 works downstream of the ESCRT‐III complex and is believed to be involved in the final scission of the budding membrane (Schöneberg *et al*., 2018).

**Figure 1:**
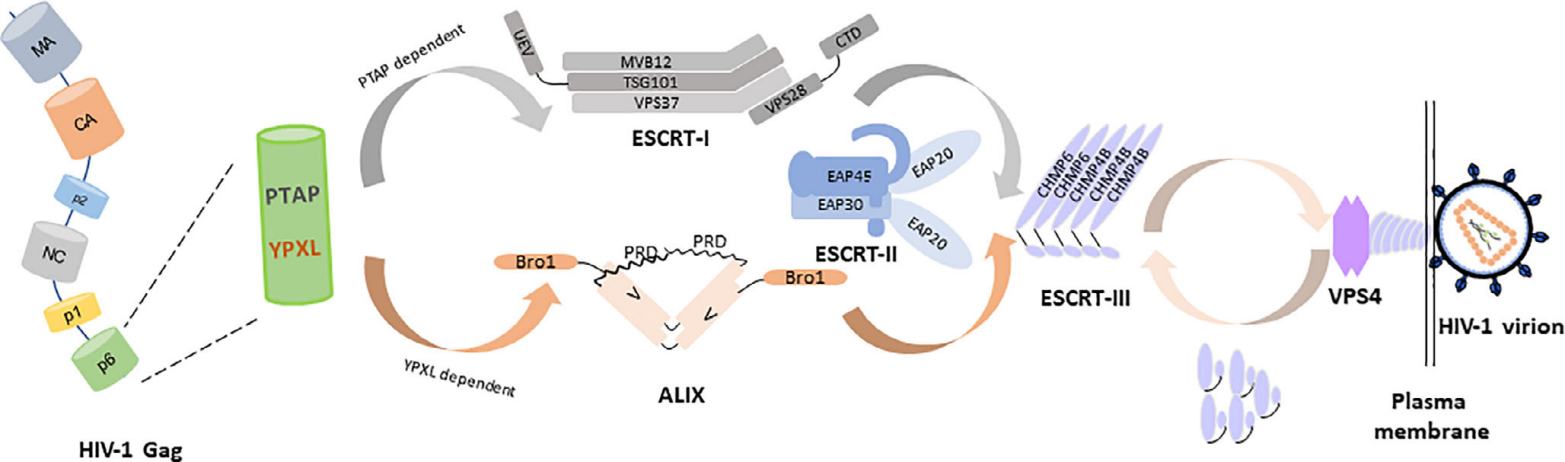
The current conceptual model for ESCRT mediated HIV‐1 budding. HIV budding initiates by specific recognition between two late motifs, located within the p6 domain of Gag, and the ESCRT complexes: the PTAP motif interacts with TSG101 of ESCRT‐I, and the YPXL motif interacts with ALIX. The requirement for the ESCRT‐II complex in this process is under debate. The two pathways converge at the recruitment of CHMP4B of the ESCRT‐III complex. The final scission event is catalysed by the AAA ATPase VPS4, which is believed to depolymerise the ESCRT‐III proteins for recycling. ESCRT‐ endosomal sorting complex required for transport; HIV‐ human immunodeficiency virus; MVB‐ multivesicular body

Several viruses have evolved the ability to hijack ESCRT complexes for their own use during productive infection (Votteler & Sundquist, 2013). Human immunodeficiency virus (HIV)‐1 is an example of this where ESCRT complexes are utilised at the late stage of its life cycle for budding (Fujii, Hurley, & Freed, 2007; Sundquist & Kräusslich, 2012; Meng & Lever, 2013; Figure 1). The major HIV structural polyprotein Gag forms a multimeric array below the plasma membrane leading to a change of membrane curvature. ESCRT complexes (ESCRT‐I and ESCRT‐associated protein ALIX) are recruited through the PTAP and YPXL motifs located in the p6 domain of Gag and initiate the budding and scission process (Demirov, Orenstein, & Freed, 2002; Garrus *et al*., 2001; Strack, Calistri, Craig, Popova, & Gottlinger, 2003; VerPlank *et al*., 2001; von Schwedler *et al*., 2003). The final cleavage involves the recruitment of VPS4 by CHMP4B through CHMP6 or ALIX. Canonically, the formation of ESCRT‐II is believed to serve as a part of the curvature sensing supercomplex at the invagination sites leading to nucleation of CHMP4B and recruitment of VPS4 for final scission to occur (Boura *et al*., 2012; Fyfe, Schuh, Edwardson, & Audhya, 2011; Lee, Kai, Carlson, Groves, & Hurley, 2015). Alternatively, the recruitment of VPS4 can be achieved directly through the ALIX‐CHMP4B pathway. In a knockdown study of CHMP6, the budding status seemed to be unperturbed (Morita*et al*., 2011) providing indirect evidence that there is an alternative route for recruiting CHMP4B that is dependent on ALIX. Indeed, ALIX has been shown to directly interact with CHMP4B (Snf7 in yeast; Kim et al., 2005; McCullough, Fisher, Whitby, Sundquist, & Hill, 2008; Wemmer *et al*., 2011), which may provide an alternative activation route for CHMP4B in sorting ubiquitinated cargo (Tang et al., 2016) and cytokinesis (Christ et al., 2016). During or soon after budding, virus maturation occurs in which a series of proteinase cleavages transform the spherical array of Gag and Gag/Pol in the immature virion into mature viral products (Bell & Lever, 2013; Sundquist & Kräusslich, 2012). The last stage of Gag cleavage where p24 is cleaved from its precursor p24‐p2 is the slowest (Pettit et al., 1994), delay of which is a hallmark for impairment of budding (Dussupt *et al*., 2011; Strack *et al*., 2003; von Schwedler *et al*., 2003). The roles of ESCRT‐I and ‐III in HIV budding are established (Garrus et al., 2001; Morita *et al*., 2011). However, it was not until recently that it was demonstrated that ESCRT‐II, which is known to play an integral part in remodelling membranes (Boura *et al*., 2012; Fyfe *et al*., 2011; Lee *et al*., 2015), is also required for efficient budding of HIV (Meng, Ip, Prestwood, Abbink, & Lever, 2015).

ESCRT‐II is composed of two copies of EAP20 and one copy each of EAP30 and EAP45 arranged in a Y‐shaped conformation (Teo, Perisic, Gonzalez, & Williams, 2004). Initial studies using siRNA knock down suggested that ESCRT‐II was not required for HIV budding in 293 T cells (Langelier *et al*., 2006), but evidence from *in vitro* studies has shown otherwise (Carlson & Hurley, 2012). Using a liposome system, work from the Hurley laboratory demonstrated with fluorophore labelled HIV Gag that it formed puncta in the presence but not the absence of ESCRT‐II and CHMP6, suggesting that ESCRT‐II is an integral part of the complex (Carlson & Hurley, 2012). It is thus possible that in the earlier studies, a small number of protein molecules survived knockdown and continued to act as a bridge linking ESCRT‐I and ‐III. Atomic force microscopy studies *in vitro* have also shown that the ESCRT‐II complex is recruited to the neck of the budding site and that the curvature of the membrane is important for ESCRT‐II attachment (Fyfe et al., 2011).

EAP45 contains an N‐terminal phosphoinositide‐binding GRAM‐like ubiquitin‐binding (Glue) domain (Slagsvold*et al*., 2005) followed by a linker H0 domain (H0) and C‐terminal winged helix domains (WH). The Glue domain is involved in recruiting the ESCRT‐I complex although its exact binding partner(s) are under debate (Im & Hurley, 2008; Langelier et al., 2006). In yeast, this domain contains two Npl4‐type zinc finger domains, NZF1 and NZF2; the former has been demonstrated to interact with VPS28 of ESCRT‐I (Teo et al., 2004). The interaction between ESCRT‐I and ‐II plays an important role in the endosomal recruitment of ESCRT‐II (Mageswaran, Johnson, Odorizzi, & Babst, 2015). However, in mammalian systems, both NZF motifs are absent. Langelier *et al*. reported that EAP45 interacts with EAP30 and TSG101, providing an alternative route for recruiting ESCRT‐I (Langelier et al., 2006). The EAP45 binding region important for this interaction was mapped to be between the C terminus of the Glue domain and the H0 linker domain. A separate study showed that EAP45 interacts with VPS28 via the same identified region (Im & Hurley, 2008). Furthermore, it was shown that the region encompassing the H0 linker within EAP45 is necessary but not sufficient for recruiting ESCRT‐I. It appears therefore that the H0 linker and possibly adjacent residues in the C‐terminal domain of Glue are important for recruiting ESCRT‐I, although the exact nature of the interaction remains elusive.

The final stage of cytokinesis, where two daughter cells are separated, is a topologically similar process to those of HIV budding and MVB formation, where ESCRT protein complexes are also recruited (Carlton & Martin‐Serrano, 2007). The role of ESCRT‐II in this process was also controversial with an early report suggesting it is not recruited to the midbody (Carlton & Martin‐Serrano, 2007) or that depletion resulted in only a minimal defect in cytokinesis (Morita*et al*., 2007; Morita *et al*., 2010). However, recent studies reported that exogenously expressed EAP45 is recruited to the midbody, and later, endogenous ESCRT‐II was also found to be localised to the same site as ALIX during this process (Christ et al., 2016; Goliand, Nachmias, Gershony, & Elia, 2014). Hence, it appears that ESCRT‐II is important in fulfilling the requirement to recruit the ESCRT‐III complex and that two pathways involved in recruiting ESCRT‐III converge. Both pathways are important in recruiting CHMP4B and VPS4 for membrane scission to occur as is also described in sorting ubiquitinated cargos into MVB (Tang et al., 2016).

Previously, we utilised a CRISPR‐Cas9 EAP45 knock out (KO) HAP1 cell line to eliminate the possibility of any residual ESCRT‐II remaining after knockdown confounding the results and to fully address the involvement of ESCRT‐II in HIV budding (Meng et al., 2015). We demonstrated that ESCRT‐II is important in efficient HIV budding but also identified additional post‐transcriptional effects of ESCRT‐II depletion. Here, we sought further mechanistic insights into the requirement of EAP45 in this process. Extending from our previous observations, we have narrowed down the effect of EAP45 in the HIV life cycle to the post‐integration stage. We expressed full length (FL) and domain‐deleted versions of EAP45 in EAP45 KO cells and quantitated viral release, allowing us to pinpoint the domain requirement of EAP45 responsible for rescue of viral budding. The effect on virus spread was assessed in a disease‐relevant CRISPR/Cas9 EAP45 KO T cell line. Our study clarifies the role of ESCRT‐II in HIV budding and reinforces that ESCRT‐II plays an integral and essential part in efficient virus production.

## 2 RESULTS

### 2.1 EAP45 acts at a post‐integration stage in HIV replication

In our previous study, either control or EAP45 KO cells were transfected with HIV WT proviral DNA, and viral protein expression was quantified by western blotting (Meng*et al*., 2015). The data showed that Gag expression is reduced in the EAP45 KO cells. It was concluded that this reduction is most likely due to a transcriptional or post‐transcriptional defect. However, it remained possible that EAP45 was also involved in the pre‐integration stage(s). To formally address this, we infected either control or EAP45 KO cells with VSV‐G pseudotyped HIV. At 48 hr post infection, the total cellular DNA was extracted and examined by quantitative real‐time polymerase chain reaction (PCR) analysis using a probe specifically targeting the integrated provirus (Brussel & Sonigo, 2003). The data show that the amount of integrated provirus in EAP45 KO cells is comparable with that in control cells (Figure S1), with no significant difference by one sample *t*‐test (*p* = .583) suggesting EAP45 is not involved at pre‐integration stages and that its function is restricted to post‐integration events.

### 2.2 EAP45 knockout reduces viral replication and secondary spread in a T cell line

**Figure 2:**
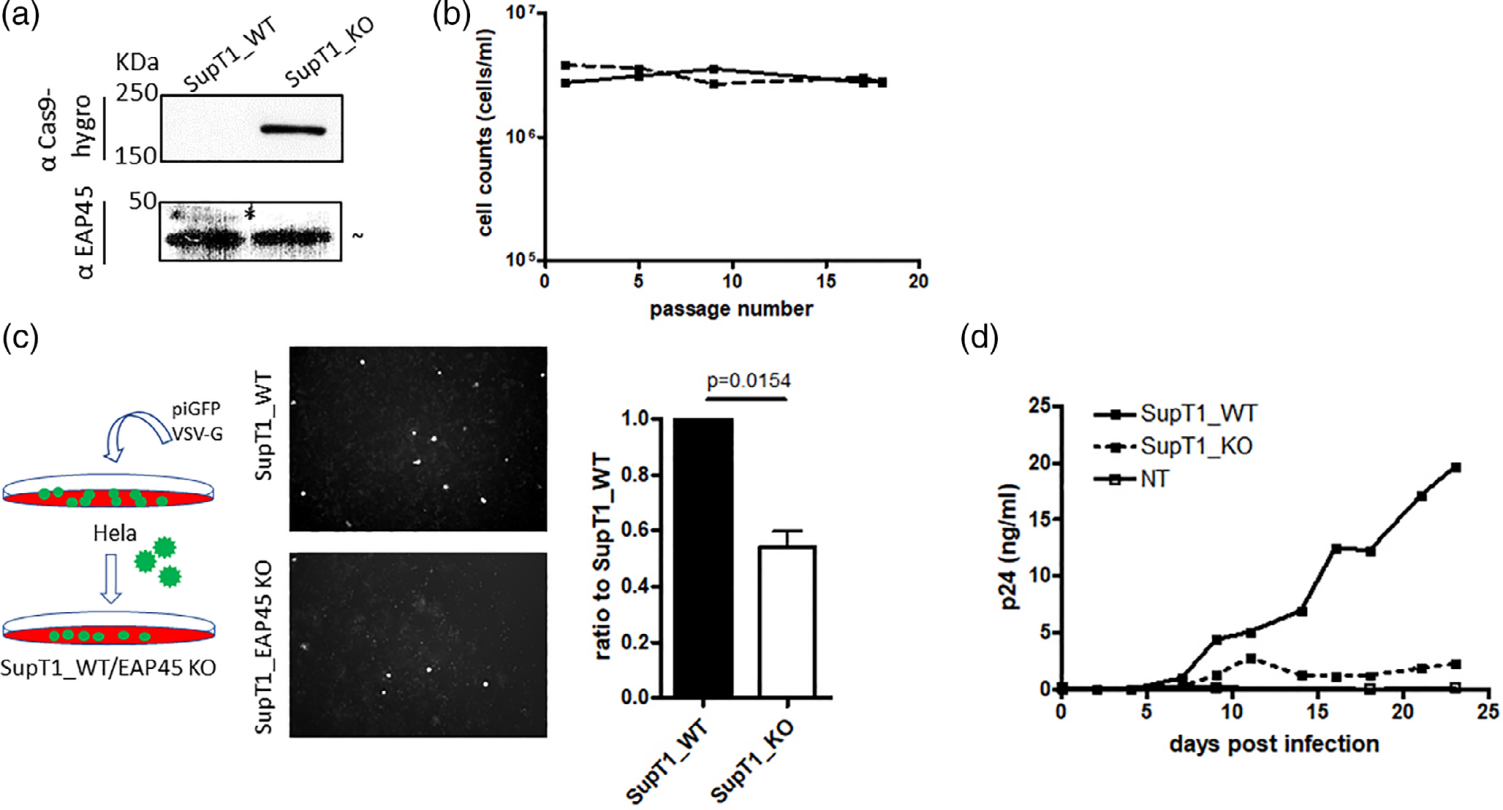
Depletion of EAP45 impairs virus spreading in SupT1 cells. (a): Western blot of the cell lysates from SupT1 WT and SupT1 EAP45 KO cells. Asterisks show the band of interest, whereas the “ ∼” indicates nonspecific products from the antibody binding on the membrane throughout this study. The size markers are shown to the left of the blot. (b): The number of SupT1 and SupT1 EAP45 KO cells was counted during 18 passages and was plotted against passage number. (c): HIV GFP virus peudotyped with VSV‐G was produced in Hela cells before transduction into either SupT1 WT or SupT1 EAP45 KO cells. The number of GFP positive cells produced from the SupT1 EAP45 KO cells was normalised against those from the SupT1 WT cells. Error bars represent the standard error of the mean from three independent experiments with a statistical significance shown by one sample *t*‐test (*p* = .0154). (d): Two million of either SupT1 WT or SupT1 EAP45 KO cells were infected with replication competent HIV BH10. The supernatants were collected every 2–3 days and quantified by p24 enzyme‐linked immunosorbent assay. The plot is representative of two experiments

We were interested to examine if the phenotype from HAP1 EAP45 KO cells was recapitulated in a disease‐relevant cell type. For this, two copies of gRNA expressors targeting exon 4 and exon 9 of the EAP45 gene together with a hygromycin fused Cas9 expressor were constructed in a transfer plasmid for lentiviral transduction into SupT1 cells. Western blot of the cell lysates from equal numbers of cells shows that EAP45 expression is successfully depleted in the Cas9‐expressing hygromycin‐resistant cells compared with WT cells (Figure 2a). Importantly, the proliferation of the cells does not seem to be impaired by EAP45 deletion over 18 passages (Figure 2b), suggesting EAP45 is redundant in cell proliferation. Single round infection in SupT1 WT or SupT1 EAP45 KO cells using HIV GFP‐expressing virus pseudotyped with VSV‐G shows a modest defect in viral gene expression possibly at a post‐transcriptional level (Figure 2c) consistent with our published results in HAP1 EAP45 KO cells (Meng *et al*., 2015). However, virus replication in SupT1 EAP45 KO cells is severely reduced to ∼10% of that seen in parental SupT1 cells (Figure 2d). These data further confirm that EAP45 plays an important role in virus spread in T cells.

### 2.3 Functional analysis of EAP45 in virus rescue

**Figure 3:**
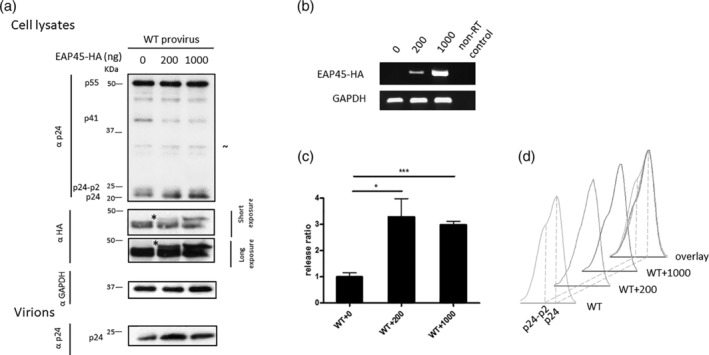
Expression of EAP45 *in trans* rescues the budding defect in HAP1 EAP45 KO cells. (a): EAP45 KO cells were transfected with WT provirus together with either 200 or 1,000 ng of HA‐tagged EAP45 expressor. Proteins from cell lysates or virions were detected by western blot using antibodies directly to HA, GAPDH, or p24. The viral products and the size markers are shown to the left of the blot. (b): The total cellular RNA was extracted followed by RT‐PCR for detection of the exogenously expressed EAP45 mRNA. Endogenously expressed GAPDH was also amplified and is shown as a loading control. (c): The release ratio is defined as the amount of p24‐associated products in purified virions divided by those in the cell lysate normalised to the level of GAPDH. The ratio is then normalised to that in the absence of EAP45. Error bars represent the standard error of the mean of four replicates from two independent experiments. Unless otherwise stated, statistical analysis was performed by using the two‐tailed student *t*‐test with significance annotated *p* ≤ .05 (*), *p* ≤ .01 (**), and *p* ≤ .001 (***) and ns (non‐significant) throughout this study. (d): The densitometric traces of p24 and p24‐p2 from (a) for each transfection condition are shown separately or in overlay to better visualise the differences in the final cleavage

We previously demonstrated that there is a decrease in virus production upon depletion of EAP45. To ascertain that this effect is specifically due to the absence of EAP45, we exogenously expressed C terminally tagged HA‐EAP45 (Meng *et al*., 2015) in HAP1 EAP45 KO cells together with the WT provirus. The production of EAP45 protein and mRNA was confirmed by immunoblotting (Figure 3a) and reverse transcription polymerase chain reaction (RT‐PCR; Figure 3b). With the increasing amount of the transfected EAP45 expressor, we observed an increase in EAP45 production both at the protein and mRNA levels that coincided with a two‐ to threefold increase in virus release (Figure 3c). This EAP45‐mediated rescue effect is specific to EAP45 KO cells because expressing EAP45 in the parental cells failed to produce any increase in virus production (Figure S2). In our previous study, we observed an accompanying defect at the last step of Gag cleavage that is classically associated with defects in viral budding (Meng et al., 2015). Interestingly, densitometric analysis of the traces of p24 and its precursor p24‐p2 revealed that this defect in cleavage was also reversed upon exogenous expression of EAP45 (Figure 3d). Our data clearly demonstrate that EAP45 is involved in efficient virus budding and that the phenotype produced by defective expression can be rescued by expressing EAP45 *in trans*.

**Figure 4:**
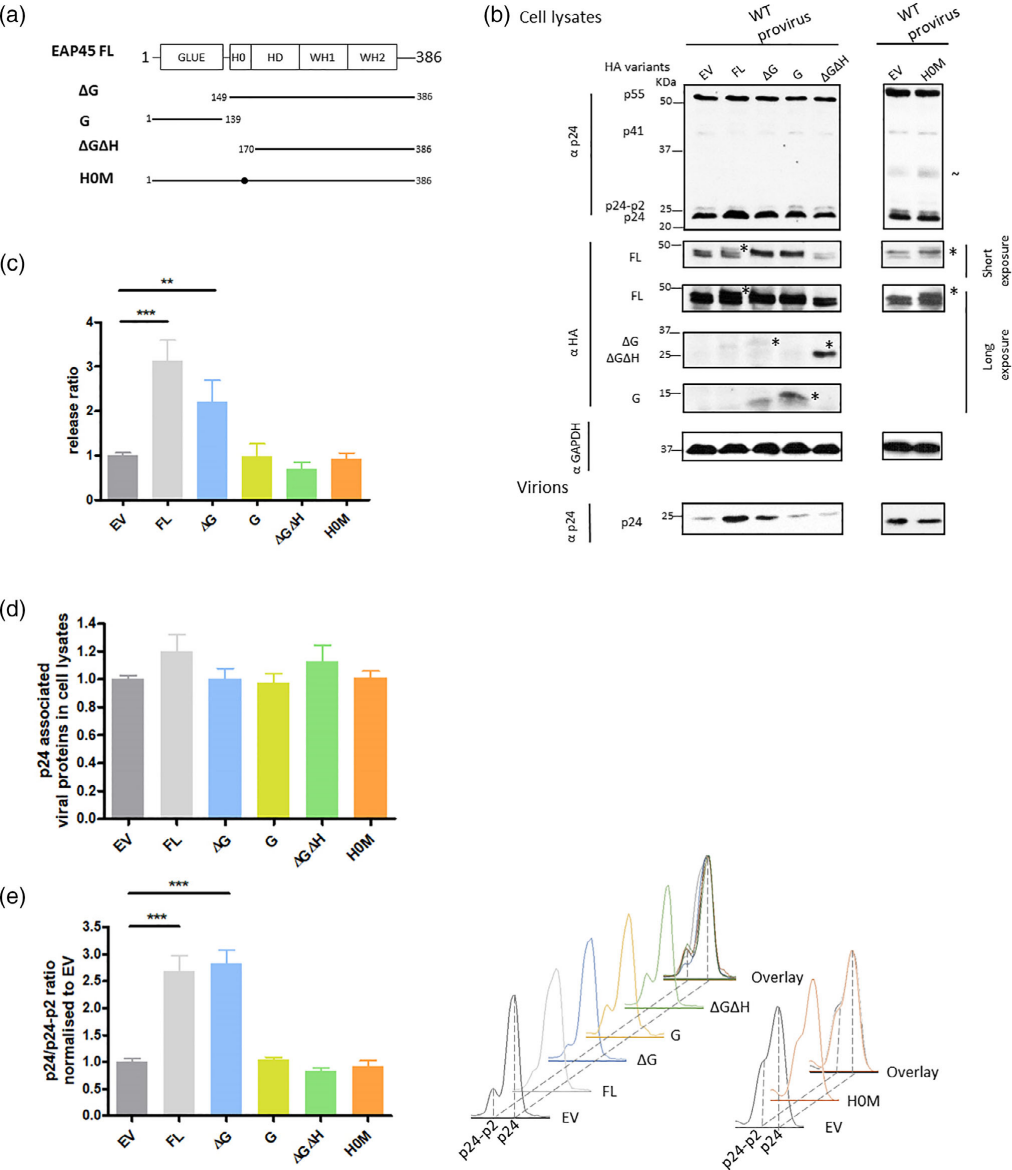
The H0 linker region in EAP45 is required for efficient rescue of HIV budding in HAP1 EAP45 KO cells. (a): Schematic diagrams of the various EAP45 variants are shown, with FL being the full‐length EAP45 expressor. Constructs are tagged with HA at the C terminus (HA tag not shown). Mutations that disrupts the putative interactions with ESCRT‐I are also introduced (H0M). The designation of each construct is shown to the left of the diagrams. (b): EAP45 KO cells were cotransfected with the WT provirus along with different EAP45 expressor mutants or empty vector (EV). Levels of proteins from cell lysates or virions were detected by immunoblotting using antibodies specific to HA, p24, or GAPDH. GAPDH was used as a loading control. Both short and long exposures are presented where necessary. (c): The virus release ratio at each condition was quantified by densitometry and normalised to that of EV as described in Figure 3. (d): Total p24‐associated viral products in the cell lysates were densitometrically quantified from Western blots and normalised to that of EV. (e): The final cleavage of Gag (p24/p24‐p2) is quantified by densitometry and normalised to that of EV. The densitometric traces of p24 and p24‐p2 for each transfection condition in (b) are shown separately or in overlay on the right. Error bars represent the standard error of the mean of five to nine replicates from at least three independent experiments

Next, we investigated the domain requirement of EAP45 (Figure 4a) for rescue of HIV budding in EAP45 KO cells using the HA‐tagged EAP45 expression constructs. The expression of each EAP45 derivative was detected in HAP1 cells by Western blot (Figure 4b). The reason for the low level of expression from the Glue domain deletion mutant (ΔG) is unknown but may be due to intrinsic structural instability caused by the presence of a flexible H0 linker region when the Glue domain is removed. This is plausible as the level of protein is restored when H0 is also removed in ΔGΔH. Upon transfecting EAP45 variants with WT provirus into EAP45 KO cells, we only observed rescue in virus release from EAP45 mutants still containing the H0 region (FL and ΔG, Figure 4c). G domain on its own and ΔGΔH failed to rescue the budding defect even though intracellular viral protein levels were unaffected (Figure 4d). Of note, ΔG could still rescue the defect efficiently despite its comparatively lower level of expression. This suggests that binding to ubiquitin is not essential in EAP45‐mediated virus release (Slagsvold et al 2005). The H0 linker region is known to be important for the ability of the Glue domain to recruit ESCRT‐I complex (Im & Hurley, 2008; Langelier *et al*., 2006). To investigate the functionality of this region in our virus rescue assay, we used a construct (H0M) where the linker region is specifically mutated to destabilise the interaction with ESCRT‐I (Im & Hurley, 2008). Mutating this region alone abolished the rescue effect completely (Figure 4c), reinforcing the notion that the H0 domain of EAP45 is critical for successful virus rescue.

Densitometric analysis of the traces of p24 and its precursor p24‐p2 showed restoration to that of WT Gag cleavage upon expressing the FL EAP45 and ΔG, but not with the G only or ΔGΔH or H0M mutants (Figure 4e). The high level of virion release with a comparatively low level of ΔG expression indicates that this mutant is particularly effective at enabling particle release. Taken together, our data show that the H0 linker region is crucial in regulating the EAP45‐mediated budding process through an evolutionarily conserved route by linking to the ESCRT‐I complex.

### 2.4 Electron microscopic examination of virus rescue

**Figure 5:**
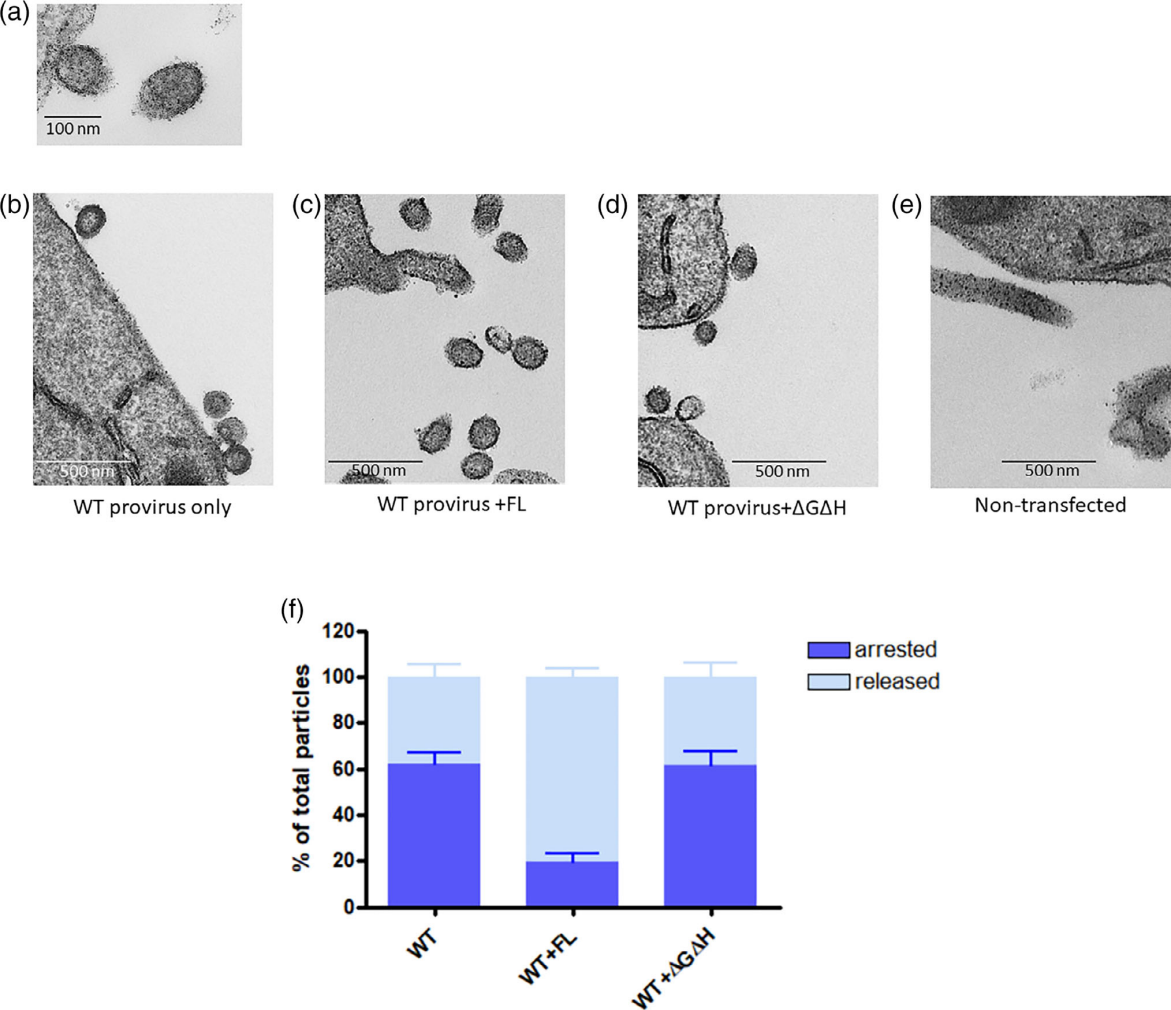
EM analysis of particles produced from transfected HAP1 EAP45 KO cells. (a and b): EM photographs show particles produced from the WT provirus‐transfected EAP45 KO cells (a and b), with EAP45 (full length [FL]; c) or with the ΔGΔH expressor (d) or nontransfected (e). Scale bar for (a) is 100 nm, whereas for (b–e), it is 500 nm. (f) Quantitation of the virus particles in each transfection condition that are either arrested at the plasma membrane or released into the extracellular compartment per field image, shown as % of total virus particles. Error bars represent the standard error of the mean of over 200 particles from a total of over 20 images

We used electron microscopy (EM) to examine the morphology of the budding virions. In HAP1 EAP45 KO cells, we observed a large number of budding viruses arrested at the plasma membrane, with stalks tethering the particles to it (Figure 5a,b, and d). Interestingly, quantitative analysis of the fold change in virus release revealed that export of virus particles increased up to threefold in the presence of exogenously expressed EAP45 (Figure 5c,f). Virions at the termini of filopodia risk being counted as “released” if the latter is not visible, so we were careful only to include virions at a distance from the plasma membrane in the released category to minimise this skewing the data. The increase in virus production is consistent with the biochemical analysis of the released virions shown by immunoblotting (Figure 4b). In a parallel experiment, we examined the morphology of released virions from ΔGΔH expressing cells, which fail to rescue the budding defect in EAP45 KO cells (Figure 5d). The arrested particles were readily seen at the plasma membrane, and the number of released particles was comparable with that in EAP45 KO cells that are not expressing exogenous EAP45 (Figure 5f). Taken together, the EM results are consistent with the data shown in Figure 4, reinforcing the notion that exogenously expressing EAP45 *in trans* rescues the budding defect and that this effect is dependent on an intact H0 linker region.

### 2.5 EAP45 functions through the ALIX pathway

It is generally believed that of the two pathways leading to activation of ESCRT‐III in HIV budding, the PTAP pathway (ESCRT‐I‐II‐III axis) is dominant for virus egress; however, cell type specificity is also documented (Demirov, Orenstein, & Freed, 2002). In HAP1 cells, we found that the YPXL pathway (ALIX axis) is preferentially used by the virus for budding (Figure S3). We reproducibly observed an even more profound reduction in virus production from the YPXL mutant than from the PTAP mutant.

If the ESCRT‐I‐II‐III axis represents one of the main pathways for HIV budding, disrupting this pathway either by removal of the PTAP late domain, where TSG101 fails to be recruited, or by depletion of EAP45 should give a similar phenotype in virus release. Importantly, this reduction in virus release should not be exacerbated in the EAP45 KO cells when transfected with the PTAP mutant. We previously observed an unexpected additional reduction (Meng et al., 2015). This suggests instead that the functions of ESCRT‐I and ‐II in this process may not completely overlap. To delineate the relationship between these two pathways, we investigated the effect of EAP45 expressed *in trans* together with either PTAP or YPXL mutated HIV.

**Figure 6:**
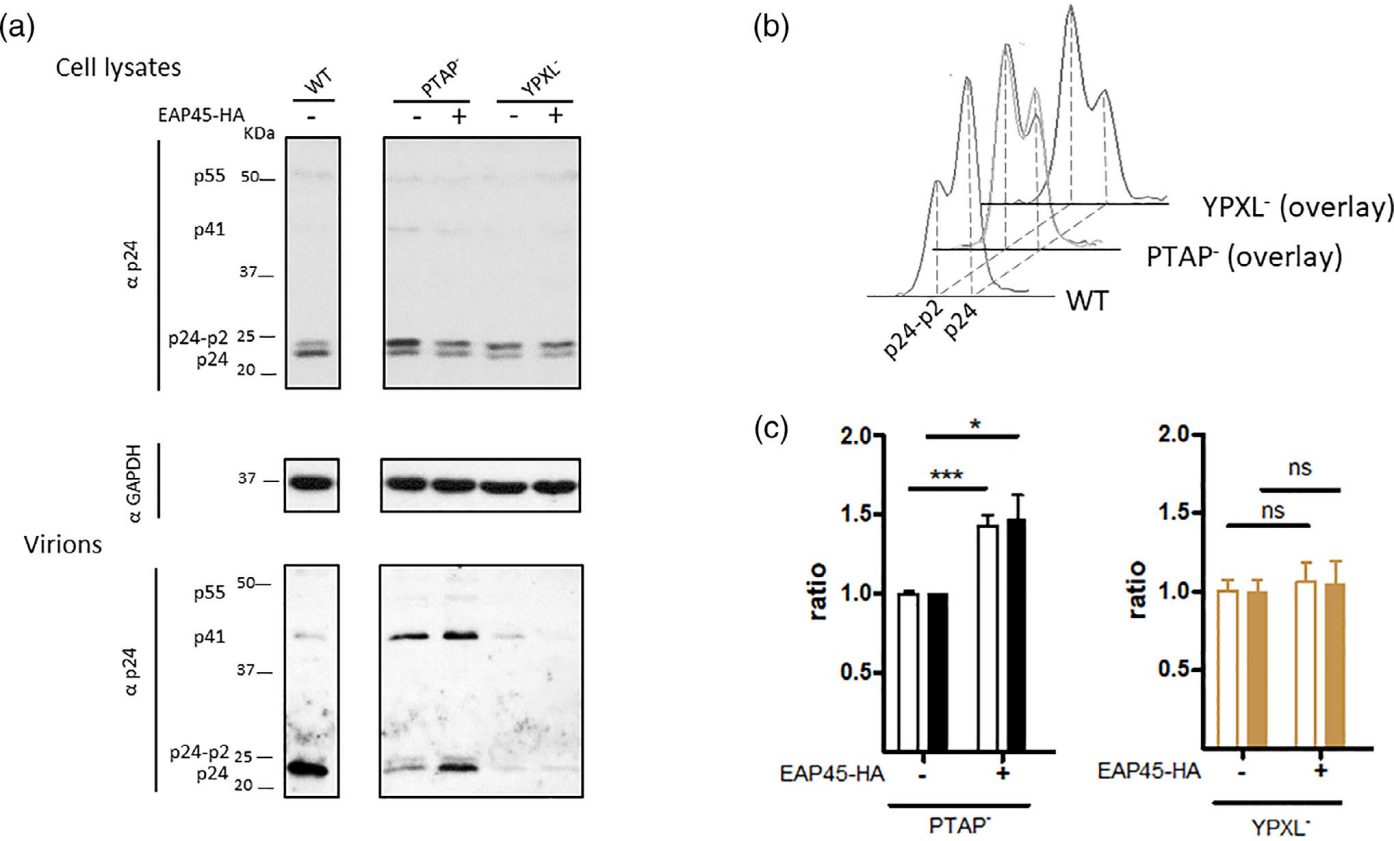
Overexpression of EAP45 in HAP1 EAP45 KO cells rescues virus release in the PTAP mutant but not in the YPXL mutant. (a): PTAP or YPXL mutant proviruses were transfected into EAP45 KO cells with or without the EAP45 expressor. WT provirus was also transfected as a control. Levels of protein from cell lysates or virions were detected by immunoblotting with antibody specific to p24. GAPDH was used as a loading control. (b): The traces of the final cleavage of Gag p24/p24‐p2 in WT, PTAP, and YPXL mutants are either shown separately (WT) or in overlay with and without EAP45 overexpression (PTAP and YPXL mutants). (c): The release (filled bars) and cleavage ratios (empty bars) for PTAP and YPXL mutants are shown, as quantitated in Figure 4. The error bars represent the standard error of the mean of more than six replicates from at least five independent experiments

**Figure 7:**
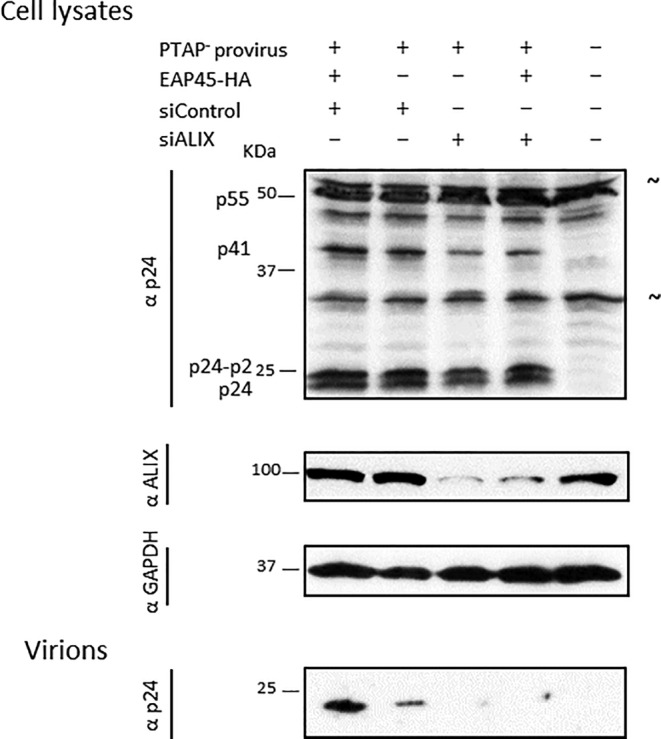
EAP45 functions through the ALIX pathway. The HAP1 EAP45 KO cells were transfected with sicontrol or siALIX followed by cotransfection of PTAP deleted proviral DNA and an EAP45 expressor. Western blots of total cell lysates and purified virions using antibodies specific to viral p24 and host proteins ALIX and GAPDH are shown. The blot is representative of three independent experiments

On providing EAP45 exogenously, the release of the PTAP mutant, strikingly, is increased, and this effect is accompanied by an increase in efficiency of the final cleavage of Gag (Figure 6). This was not observed from the YPXL mutant, suggesting that the increase of virus production and enhancement of Gag cleavage efficiency are both dependent on accessibility to ALIX. This effect of EAP45 in rescuing the budding defect of a PTAP mutant is highly reminiscent of what is seen when overexpression of ALIX rescues release of a PTAP mutant (Fisher et al., 2007; Usami, Popov, & Gottlinger, 2007). This strongly implies that EAP45 and ALIX may at least partially function through the same pathway. To further investigate this possibility, ALIX was knocked down by siRNA in EAP45 KO cells, followed by cotransfection of an EAP45 expressor and a PTAP mutant provirus. The total cell lysates and virions from the supernatant were assayed and quantified by Western blot (Figure 7). As expected, the amount of released virions is further reduced when ALIX is knocked down. Upon expression of EAP45, in contrast to the increased virus release from the control siRNA treated cells, enhancement in virus production is abolished when the endogenous level of ALIX is knocked down. These findings strongly implicate ALIX in mediating the function of EAP45 in virus rescue.

### 2.6 Release of virus‐like particles in EAP45 KO cells is independent of EAP45

VLPs (virus‐like particles) are produced from Gag only expressors lacking late domains, and the level of their release is comparable with that of a WT Gag expressor at the steady state (Bendjennat & Saffarian, 2016). Although this may suggest that ESCRT is not required in VLP production, the release of such VLPs is in fact dependent on ESCRT complexes as overexpression of the dominant negative form of VPS4 abolished their release (Bendjennat & Saffarian, 2016). This implies that factors other than p6 domains may contribute to this budding process (Gan & Gould, 2012; Kaye & Lever, 1996; Norgan et al., 2012). More recently, it was observed that, even though the budding is similar in those mutants, the release kinetics of VLP from a Gag expressor bearing late domain mutations is slower with a delay of up to an hour (Bendjennat & Saffarian, 2016).

**Figure 8:**
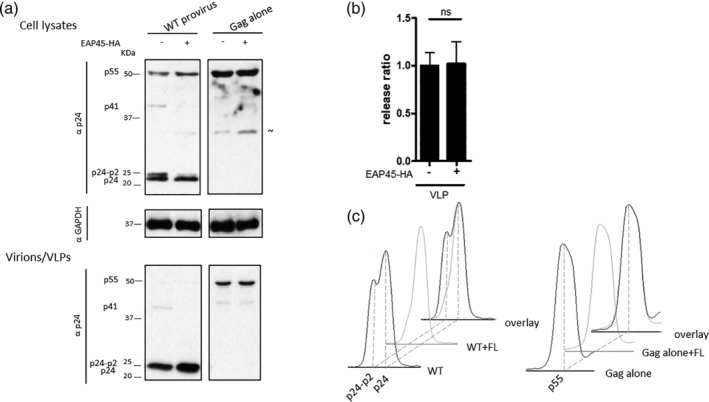
Virus‐like particle (VLP) levels produced from a Gag expressor alone are not altered upon overexpression of EAP45 in HAP1 EAP45 KO cells. (a): EAP45 KO cells were transfected with WT provirus or a Gag expressor in the absence or presence of EAP45. Levels of protein from cell lysates or purified virions were detected by western blot using antibodies specific to p24 or GAPDH. (b): The VLP release ratio at each condition was quantified by densitometry and normalised to that in the absence of EAP45 expressor. The error bars represent the standard error of the mean of four replicates from three independent experiments. (c): The densitometric traces of p24 and p24‐p2 for WT provirus or p55 for a Gag expressor alone are shown separately or in overlay

Having demonstrated that EAP45 is required for efficient HIV budding where Gag/Pol is intact, we were curious to know if the rescue effect observed was still evident in a sole Gag expressor transfected into EAP45 KO cells at the steady state. Upon transfection of a Gag only expressor, the quantity of VLPs released did not increase in the presence of EAP45 (Figure 8a,b). Our observation of a minimal effect in Gag VLP release but a greater effect with intact virus is surprising. However, it suggests that the presence of Gag/Pol may be necessary for the rescue effect as observed in Figure 3.

**Figure 9:**
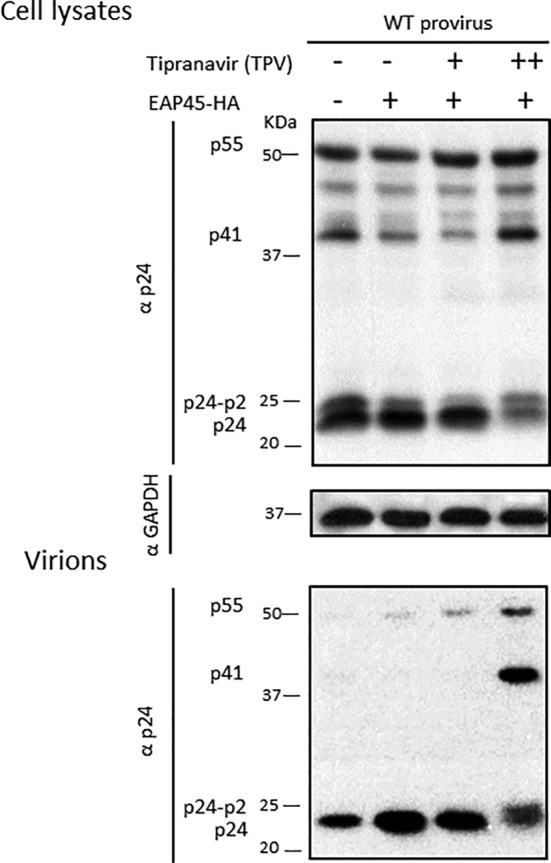
The rescue effect of EAP45 in HAP1 EAP45 KO cells is independent of a functional viral protease. EAP45 KO cells were transfected with WT provirus in the presence or absence of the EAP45 expressor and various amounts of TPV (+: 200 nM or ++: 500 nM). Cell lysates and purified virions were immunoblotted with antibodies to either p24 or GAPDH. The blot is representative of two independent experiments

Gag/Pol once packaged is cleaved to produce reverse transcriptase, integrase, and protease, although how autolytic cleavage is initiated is still unclear. Loss of protease has been proposed as one of the key determinants explaining the observation of the late budding effects. One possible interpretation from Figure 8 would be that the requirement for Gag/Pol is to supply an activated protease for successful restoration of virion release by EAP45. To explore this, we examined budding in the presence of the protease inhibitor tipranavir (TPV). The addition of TPV produced the expected dose‐dependent impairment of Gag processing (Figure 9 & S4). Interestingly, exogenous expression of EAP45 in KO cells still rescued virus release in the presence of the drug (Figure 9). This suggests that a functional viral protease is not a prerequisite for the enhanced budding mediated by EAP45.

### 2.7 The final cleavage of Gag correlates with virus release

**Figure 10:**
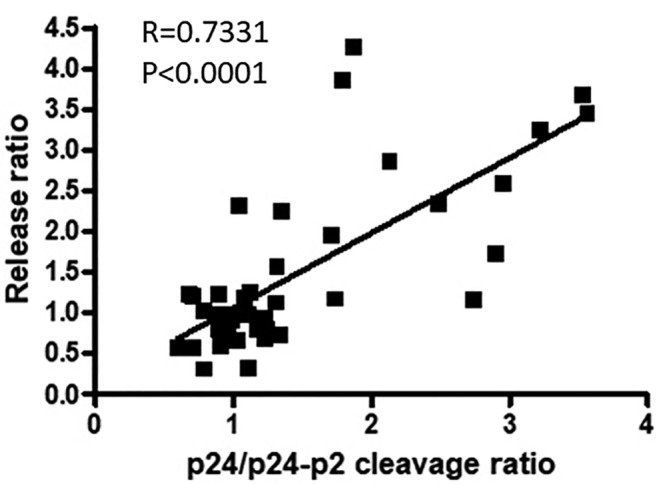
The final cleavage of Gag correlates with virus release upon expressing EAP45. Data from 48 replicates of both WT provirus and PTAP mutant in the absence or presence of EAP45 were pooled and analysed by Pearson correlation analysis. A significant correlation of released virions with the final Gag cleavage was detected (*r* = .7331; *p* < .0001)

Failure of the cleavage of p24 from p24‐p2 by the viral protease is regarded as the signature defect associated with impairment of ESCRT mediated viral budding (Demirov, Ono, Orenstein, & Freed, 2002; Strack *et al*., 2003; von Schwedler *et al*., 2003). ALIX has been shown to rescue this cleavage defect when expressed with a PTAP mutated virus. Our results now demonstrate that this cleavage defect can also be reversed by restoration of EAP45 in the EAP45 KO cell line. To test whether this increase of Gag cleavage correlates with an increase in virus release, we pooled all the data from the expression studies including the PTAP mutant and quantified the release and the corresponding final cleavage by covariance analysis. Remarkably, we found that the release and final cleavage rate of p24/p24‐p2 are significantly correlated (Pearson correlation coefficient .7331; Figure 10). This is indicative that the two processes are strongly linked.

## 3 DISCUSSION

During or soon after HIV buds from the cell, autocleavage of Gag/Pol occurs to produce activated viral protease, which leads to sequential proteolytic cleavage of Gag and Gag/Pol (Bell & Lever, 2013; Sundquist & Kräusslich, 2012). The timing of this process is believed to be critically important to maintain proper virion maturation and virion infectivity. Delayed budding caused by late domain or NC mutation leaves the virus budding neck open allowing the activated protease to diffuse back into the cytosol (Bendjennat & Saffarian, 2016; Chamontin *et al*., 2015). This loss of protease is thought to be instrumental in producing the maturation defect observed in budding defective viruses. Depletion of EAP45 gives rise to viruses in which the last step of Gag cleavage is also defective, which is the first indication that EAP45 plays a role at the late stage of the HIV life cycle (Meng *et al*., 2015). This defect is accompanied by a defect in virus release, and we show that it can be successfully restored when EAP45 is expressed exogenously (Figure 3). The increase in virion release triggered by EAP45 rescue is, however, not dependent on the presence of an active protease (Figure 9), suggesting the rescue effect of EAP45 in EAP45 KO cells is independent of virion maturation, and that budding neck closure precedes the activation of the viral protease. Our data are consistent with the notion that in EAP45 KO cells, recruitment of EAP45 is required to restore defective budding kinetics and to facilitate the sealing of the budding neck so that the activated protease is confined within the budding virions to allow Gag maturation to proceed (Bendjennat & Saffarian, 2016; Chamontin *et al*., 2015). The significant correlation between the final cleavage of Gag and virion release also supports this notion (Figure 10). Interestingly, this rescue of budding is only observed when Gag/Pol is present (Figure 8). This suggests that EAP45 (and possibly the entire ESCRT‐II complex) acts like TSG101 and ALIX at the early stage of the budding process and is more effective at packaging a large cargo. This necessity to package Gag/Pol in ESCRT‐II‐mediated budding provides an alternative explanation for an earlier study where VLP release was found to be unaltered when ESCRT‐II was depleted (Pincetic, Medina, Carter, & Leis, 2008). Our data suggest that the virus uses an additional safeguard to favour production of infectious virions, regulated by early ESCRT proteins facilitating virion maturation and acting as a checkpoint to ensure Gag/Pol inclusion into the virus; failure of autoprocessing of Gag and Gag/Pol and consequential absence of protease would otherwise render the virus noninfectious.

The lipid targeting ability of ESCRT‐II at the plasma membrane is not fully understood. However, it is possible that the ESCRT‐II‐CHMP6 complex senses the negative membrane curvature as Gag multimerises at the plasma membrane in the presence of EAP45. The accumulation of such complexes could lead to further nucleation of CHMP4B for final scission to occur. However, the membrane curvature sensing ability of these complexes is necessary but not sufficient for a successful budding event because our evidence shows that the H0 linker region in EAP45, which is known to bind ESCRT‐I, is also required; only mutants containing H0 rescue the budding defect in EAP45 KO cells (Figure 3). The H0 linker region both recruits the ESCRT‐I complex and stabilises the ESCRT‐II complex for recruiting CHMP6 for viral budding. The evidence thus points to ESCRT‐II bridging ESCRT‐I and ‐III complexes in this system as it does in others; mutating the H0 linker region alone completely abolishes the effect in rescue, and Gag cleavage remains defective (Figure 3). Our quantitative EM analysis also shows that the budding particles remain tethered at the plasma membrane when H0 is absent, whereas the particle production is dramatically increased in the presence of intact EAP45 (Figure 5f). Intriguingly, the Glue domain therefore seems to play an inhibitory role for the activation of ESCRT‐II as upon removal of this region; the virus rescue is almost as efficient as that achieved by the FL protein (Figure 4). An autoinhibitory role within members of ESCRT is not uncommon (McCullough *et al*., 2008) and has been proposed also for ESCRT‐II for its role in MVB sorting in yeast (Mageswaran *et al*., 2015). However, activation of ESCRT‐II in MVB sorting is slightly different from that in HIV budding, as the removal of the Glue domain is not sufficient to rescue the cargo sorting defective phenotype; a mutation is additionally required at its C terminus.

We observed that expression of EAP45 rescues virus release of a PTAP mutant, accompanied by an increased efficiency of the final cleavage step of Gag (Figure 6). This mimics the phenotype of overexpression of ALIX in the presence of a PTAP mutant (Fisher *et al*., 2007; Usami *et al*., 2007). This increase in virus release fails to occur when ALIX is also knocked down in EAP45 KO cells (Figure 7), suggesting EAP45 rescues virus release through the ALIX pathway. Consistent with this model, overexpression of EAP45 fails to increase viral release of the YPXL mutant, suggesting ESCRT‐II is dispensable in this regard possibly due to a direct interaction between VPS28 of ESCRT‐I and CHMP6 of ESCRT‐III providing an alternative bypass (Pineda‐Molina *et al*., 2006). ESCRT‐II may, however, function through ALIX, mediated by the interaction between ALIX and TSG101 (Langelier *et al*., 2006; Strack et al., 2003; von Schwedler *et al*., 2003). Binding of YPXL to the central V domain of ALIX releases the sequestered C‐terminal domain of ALIX and transforms ALIX from a closed to an open position (Zhou et al., 2008). This conformational change provides an additional binding site for TSG101. The recruitment of TSG101 in this process may further recruit downstream CHMP4B through binding to EAP45 of the ESCRT‐II complex. This logically explains why rescue can only be observed by expression of EAP45 with the PTAP mutant but not with the YPXL mutant (Figure 9). The same network of interactions has recently been clarified in cytokinesis whereby the role of ESCRT‐II is to aid recruitment of CHMP4B for abscission (Christ *et al*., 2016). However, an alternative possibility, which is not mutually exclusive, is that overexpression of EAP45 enhances the interaction of Gag and ALIX probably through the NC domain, and this enhanced interaction leads to an acceleration in CHMP4B polymerisation (Sette *et al*., 2016). If so, it is tempting to speculate that the accessibility of ALIX is important in nucleating CHMP4B at the budding site to lower the threshold that is needed for an efficient budding event to occur. Alternatively, there may be an as yet unidentified factor functioning through this pathway. The findings that CHMP4B and VPS4 are packaged into VLP upon overexpression in the absence of PTAP and YPXL domains (Bendjennat & Saffarian, 2016) suggest that other factors, for example ESCRT‐II, may also be involved directly in this process independent of late domains.

**Figure 11:**
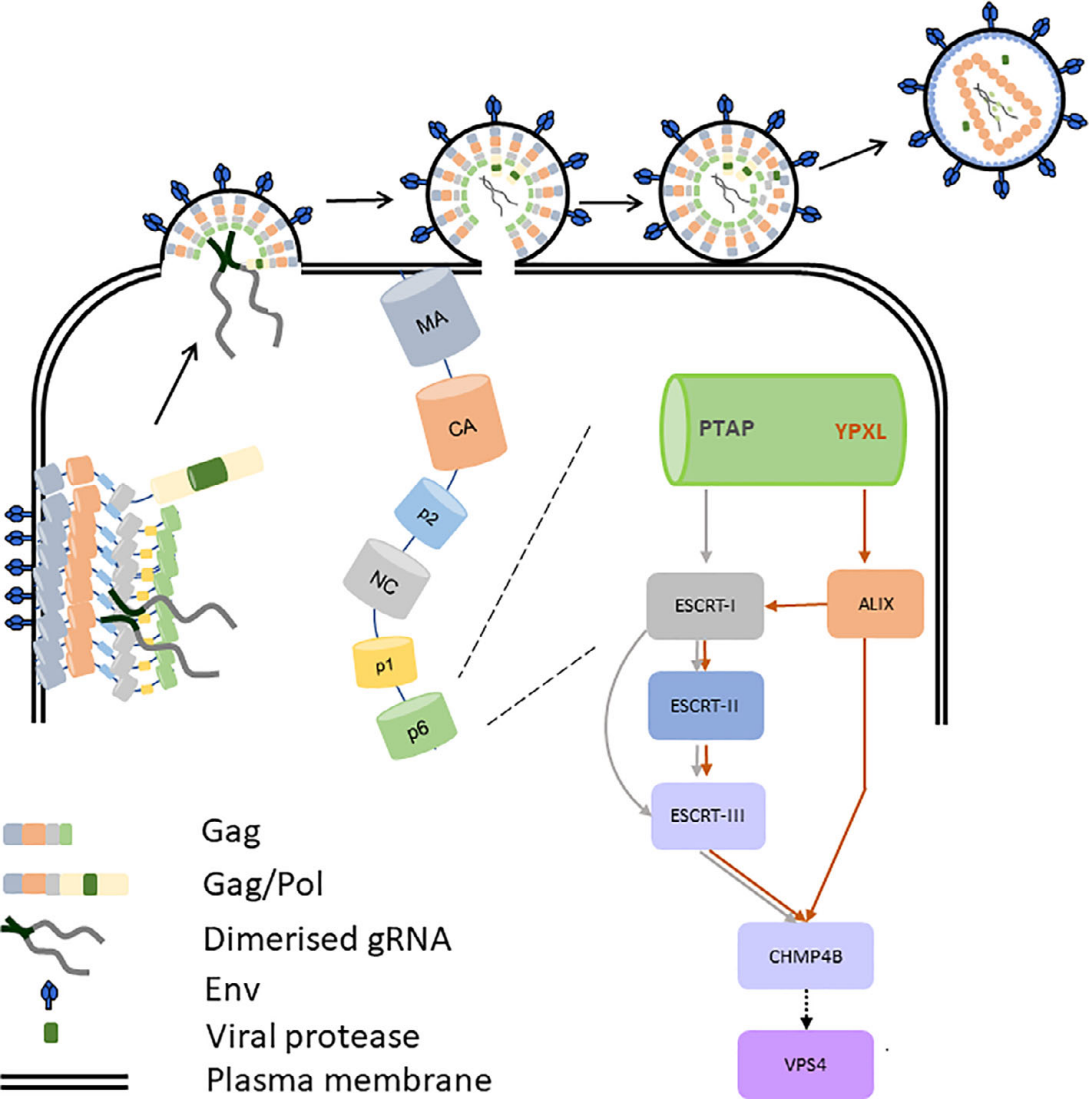
Cartoon representation of the pathways in HIV budding. Multimerisation of Gag with Gag/Pol and the genomic RNA at the plasma membrane causes the change in membrane curvature. Two late domain motifs (PTAP and YPXL) located within the p6 domain of Gag recruit subunits of ESCRT‐I and/or ALIX to the budding site. ESCRT‐II forms as a part of membrane sensing supercomplex and functions through the ALIX pathway. The two pathways converge at the recruitment of CHMP4B and VPS4 for the final scission. ESCRT‐II (together with other early ESCRT proteins) is also a prerequisite for Gag/Pol recruitment. Once the budding neck is sealed, HIV virus‐mediated protease cleavage occurs transforming immature particles to mature infectious virions

Our data confirm that ESCRT‐II is intimately involved in HIV budding and expand on our previous findings to show that the same holds true in a physiologically relevant T cell line. We show that ESCRT‐II functions at the early stage of virus export and is involved in efficient HIV budding through its canonical role of bridging ESCRT‐I and ‐III complexes (Figure 11). We propose a model whereby ESCRT‐I interacts with and activates ESCRT‐II though the C‐terminal Glue and H0 linker domains in EAP45 which then stabilises the complex for recruiting CHMP6. ESCRT‐II is more efficient at enabling the enhancement in virus release in the presence of Gag/Pol to ensure inclusion of the viral protease for processing of Gag and other Gag/Pol polyproteins. ALIX is required most likely due to the necessity of increasing the local concentration of CHMP4B at the budding site through both the canonical ESCRT‐I‐II‐CHMP6 pathway and by direct recruitment of CHMP4B. The evolutionarily conserved ESCRT‐I‐II‐CHMP6 complex, therefore, appears to play a synergistic role with the ALIX pathway to permit efficient budding of infectious HIV particles, showing remarkable parallels to the cellular processes of cytokinesis and sorting of ubiquitinated cargos.

## 4 EXPERIMENTAL PROCEDURES

### 4.1 Cells and plasmids

All cells used in this study were grown at 37°C in 5% CO_2_ and have been described previously (Meng *et al*., 2015). pBH10ΔBglII‐WT and pCMV‐VSV‐G, pBH10ΔBglII‐YPXL and pBH10ΔBglII‐PTAP mutants, pEF‐EAP45‐HA and its HA‐tagged mutants cloned originally from pGEX4T1‐EAP45 (Bowers*et al*., 2006) were reported previously (Meng*et al*., 2015). A sole Gag expressor (HVPgagpro‐) was also described previously (Kaye & Lever, 1996). piGFP (GFP is inserted between MA and CA) was described previously (Hubner *et al*., 2007). A transfer plasmid consisting of gRNA expressors targeting exon 4 and exon 9, respectively, and a hygromycin fused Cas9 expressor was constructed. pMDLg/pRRE and pRSV‐Rev were both obtained from Addgene.

### 4.2 Quantitative RT‐PCR for quantitation of integrated proviruses

A total of 293 T cells were transfected with pBH10ΔBglII‐WT and pCMV‐VSV‐G expressing plasmids using TransIT LT1 (Mirus) as described previously (Meng *et al*., 2015). At 2‐days post transfection, the supernatant was harvested, and virus infectivity was measured in a TZmbl infectivity assay. The same amount of pseudotyped HIV WT viruses (10 ng p24) were used to infect an equal number of either control or EAP45 KO cells. After the 2‐day incubation at 37°C, the supernatant was removed before the total DNA was extracted using a Qiagen blood and tissue extraction kit (Qiagen). The integrated proviral DNA was quantified by *Alu* PCR based on a published protocol (Brussel & Sonigo, 2003). In a typical quantitative RT‐PCR analysis, the Ct value from non‐*Alu* primers reaction was subtracted from the Ct value of a reaction where *Alu* primers were added. Relative quantitation (ΔΔCt) of the amount of integrated provirus in EAP45 KO cells compared with that of control cells was measured.

### 4.3 Construction of SupT1 EAP45 KO cells

The lentivirus that packages two gRNA targeting the EAP45 gene was produced in 293 T cells. The hygromycin selection media was applied 3 days post transduction in SupT1 cells. Western blot analyses of the cell lysates were used to verify the knock out of EAP45 and the expression of Cas9‐hygromycin fusion protein using antiEAP45 (Malerod, Stuffers, Brech, & Stenmark, 2007) and antiCas9 (Takara). For a single round infection, HIV‐GFP virus pseudotyped with VSV‐G was used to transduce either SupT1 WT or SupT1 EAP45 KO cells. Two days post transduction, the GFP positive cells were counted. For a multiround infection, 40 ng of p24 of the replication competent BH10 viruses produced in 293 T cells was used to infect 2 million of either SupT1 WT or SupT1 EAP45 KO cells. The supernatant was collected every 2–3 days, and the production of viral p24 was quantified by p24 enzyme‐linked immunosorbent assay.

### 4.4 RT‐PCR

Total cellular RNA was extracted from EAP45‐HA expressing cells using a Qiagen RNA extraction kit (Qiagen). A set of primers specific for exogenously expressed HA‐tagged EAP45 mRNA was used in an RT‐PCR reaction. As a control, a set of GAPDH primers was used for detection of the endogenously expressed GAPDH. Primer sequences are available upon request.

### 4.5 Virus rescue assay

One million control or KO cells were transfected in each well of a six‐well plate using TurboFectin 8.0 (Origene). A total amount (1333 ng) of either pBH10ΔBglII‐WT, pBH10ΔBglII‐YPXL, or pBH10ΔBglII‐PTAP together with pCMV‐VSV‐G was transfected into control or EAP45 KO cells in the presence of 200 ng of either HA‐tagged FL EAP45, EAP45 variants, or empty vector (EV).

### 4.6 VLP production assay

A total amount of 200 ng of either pBH10ΔBglII‐WT or HVPgagpro, together with pCMV‐VSV‐G, in the presence of 45 ng EAP45 FL expressor or EV, was transfected into 70% confluent EAP45 KO cells in each well of a 24‐well plate using TurboFectin 8.0.

### 4.7 Viral protease inhibition assay

Either control or EAP45 KO cells in a 24‐well plate were transfected with a total amount of 200 ng of pBH10ΔBglII‐WT and pCMV‐VSV‐G expressing plasmid in the absence or presence of Tipranavir (200‐ or 500‐nM final concentration; NIH AIDS reagent). In some transfections, an EAP45‐HA expressor or EV was also cotransfected to examine the rescue effect of EAP45 during TPV treatment.

### 4.8 Immunoblotting

Typically, 2 days post transfection, supernatants were harvested for virion purification by Optiprep ultracentrifugation, and monolayers were lysed in cell culture lysis buffer for immunoblotting. Cell lysates and purified virions were electrophoresed by SDS‐PAGE followed by transfer to a nitrocellulose membrane. Blots were incubated with the primary antibody followed by incubating with secondary antibody before detection with either ECL (Promega) or ECL prime (GE healthcare). The primary antibodies used in this study are anti‐p24 (NIBSC), anti‐GAPDH (Abcam), anti‐HA (Thermofisher), anti‐ALIX (Cell signalling), and anti‐TSG101 (Abcam), and secondary antibodies used were goat anti‐mouse IgG‐HRP (cell signalling) and goat anti‐rabbit IgG‐HRP (Thermofisher). Densitometric analysis of the target bands was done by ImageJ (NIH) with traces of p24 and p24‐p2 highlighted, where needed, to show the differences in the final cleavage product.

### 4.9 Transmission electron microscopy of transfected cells

Transmission electron microscopy was performed as described previously with a slight modification (Meng *et al*., 2015). The EAP45 KO cells were transfected with pBH10ΔBglII‐WT provirus and pCMV‐VSV‐G plasmid in the presence of EAP45‐HA (FL) or ΔGΔH‐HA expressing constructs. At 48‐hr post transfection, the supernatant was removed, and the monolayer of cells was washed and fixed. The cells were scraped and collected by centrifugation before being embedded and thin‐sectioned. The thin‐sectioned samples were mounted on an EM grid and visualised using a Tecnai G2 electron microscope.

### 4.10 siRNA knock down

siRNA knock down was performed essentially as described previously (Meng et al., 2015). Briefly, 2 × 10^5^ EAP45 KO cells were transfected by TransIT‐TKO (Mirus) with one dose of siRNA (siControl and siALIX; Thermofisher) on Day 1. A second dose of siRNA was transfected together with the total amount of 200 ng of pBH10ΔBglII‐PTAP and pCMV‐VSV‐G expressing plasmid with either 45 ng of EAP45‐HA expressor or EV. On Day 4 post transfection, the supernatant was harvested and virions purified as described above. The monolayer was lysed with 1× cell culture lysis buffer before immunoblotting for viral or host proteins.

## CONFLICT OF INTERESTS

The authors declare that they have no financial or other competing interests.

## Supporting information


**Figure S1 EAP45 is not required at the pre‐integration stages of the HIV life cycle**. HIV‐1 pseudotyped viruses were used to infect either control or HAP1 EAP45 KO cells. At 48 hours post infection the total DNA was extracted and integrated proviral DNA was quantified by quantitative real‐time PCR and normalised against that of control cells. The error bars represent the standard error of the mean from three independent experiments.

**Figure S2 The virus rescue mediated by EAP45 is only observed in the HAP1 EAP45 KO cells but not control cells**. Either control or EAP45 KO cells were co‐transfected with WT provirus and increasing amounts of EAP45 expressor. The cell lysates and virions were assayed by western blot. The blot is representative of two independent experiments.

**Figure S3 The YPXL motif is important in virus budding in HAP1 cells**. Either WT, PTAP or YPXL mutated proviruses were transfected into HAP1 control cells. The total cell lysates and virions purified from supernatant were analysed by western blot using antibodies specific to p24. GAPDH was used as a loading control. The blot is representative of three independent experiments.

**Figure S4 TPV inhibits virion maturation**. Control cells were transfected by WT provirus in the absence of or increasing amounts of TPV (+: 200 nM or ++: 500 nM). The cell lysates and supernatants were assayed by western blot using antibodies specific to p24. GAPDH was used as a loading control. The blot is representative of two independent experiments.
