# Supplementary figures and images for: ESCRT‐II functions by linking to ESCRT‐I in human immunodeficiency virus‐1 budding
Source: Cell Microbiol. 2020 Feb 14;22(5):e13161. doi: 10.1111/cmi.13161 (PMC7187348; doi:10.1111/cmi.13161)

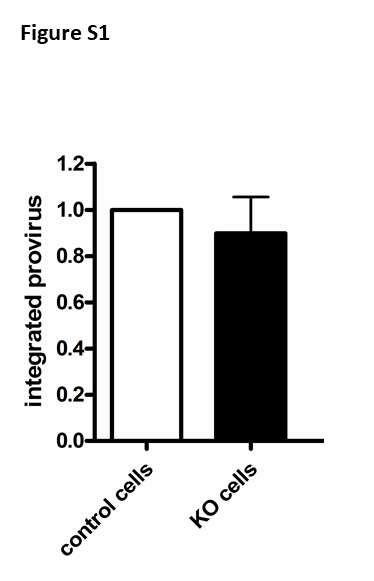

Supplement: Supplementary file 1 — Figure S1 EAP45 is not required at the pre‐integration stages of the HIV life cycle. HIV‐1 pseudotyped viruses were used to infect either control or HAP1 EAP45 KO cells. At 48 hours post infection the total DNA was extracted and integrated proviral DNA was quantified by quantitative real‐time PCR and normalised against that of control cells. The error bars represent the standard error of the mean from three independent experiments. [file CMI-22-e13161-s001.tif]

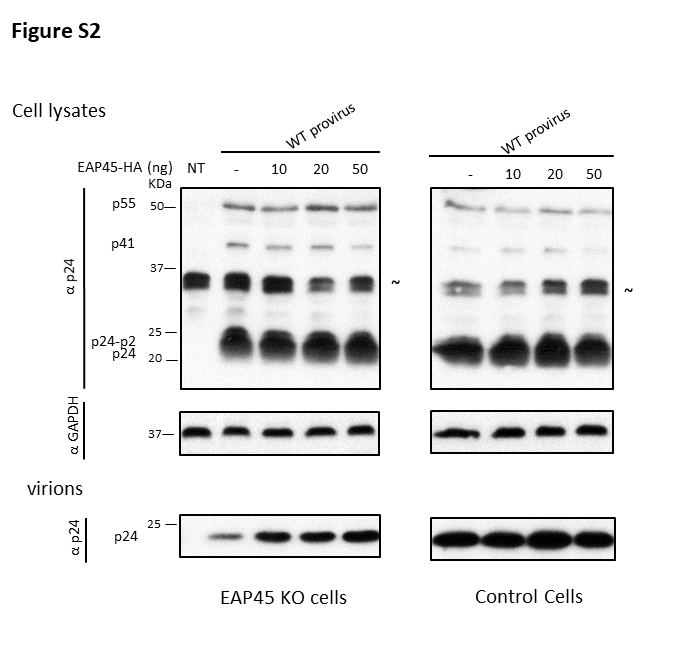

Supplement: Supplementary file 2 — Figure S2 The virus rescue mediated by EAP45 is only observed in the HAP1 EAP45 KO cells but not control cells. Either control or EAP45 KO cells were co‐transfected with WT provirus and increasing amounts of EAP45 expressor. The cell lysates and virions were assayed by western blot. The blot is representative of two independent experiments. [file CMI-22-e13161-s002.tif]

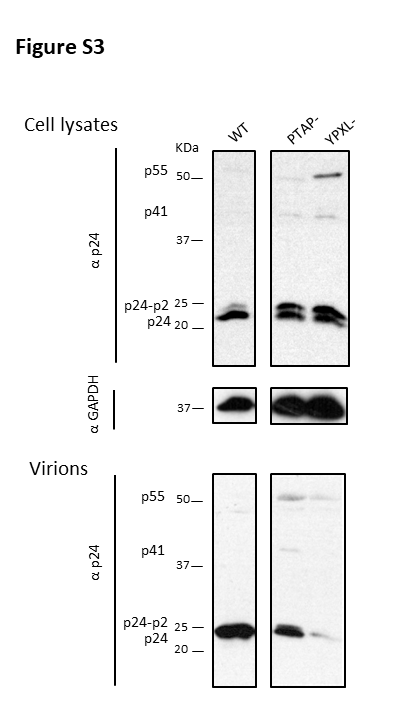

Supplement: Supplementary file 3 — Figure S3 The YPXL motif is important in virus budding in HAP1 cells. Either WT, PTAP or YPXL mutated proviruses were transfected into HAP1 control cells. The total cell lysates and virions purified from supernatant were analysed by western blot using antibodies specific to p24. GAPDH was used as a loading control. The blot is representative of three independent experiments. [file CMI-22-e13161-s003.tif]

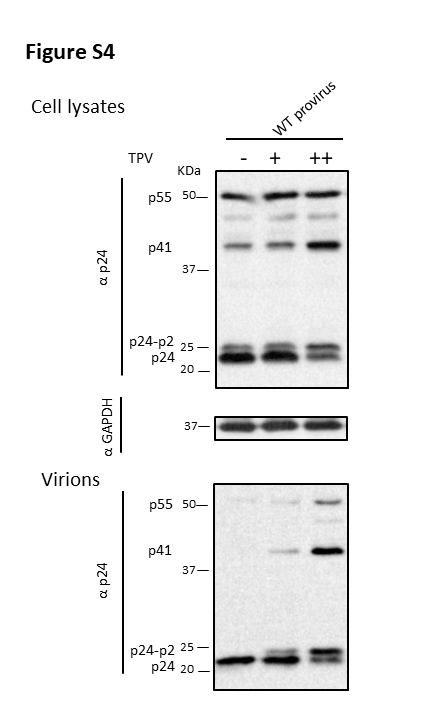

Supplement: Supplementary file 4 — Figure S4 TPV inhibits virion maturation. Control cells were transfected by WT provirus in the absence of or increasing amounts of TPV (+: 200 nM or ++: 500 nM). The cell lysates and supernatants were assayed by western blot using antibodies specific to p24. GAPDH was used as a loading control. The blot is representative of two independent experiments. [file CMI-22-e13161-s004.tif]
